# STAT Signature Dish: Serving Immunity with a Side of Dietary Control

**DOI:** 10.3390/biom15040487

**Published:** 2025-03-26

**Authors:** Hozaifa Metwally

**Affiliations:** Laboratory of Immune Regulation, The World Premier International Research Center Initiative (WPI) Immunology Frontier Research Center, Osaka University, Osaka 565-0871, Japan; hozaifa1@ifrec.osaka-u.ac.jp

**Keywords:** STAT, signaling, immunity, diet

## Abstract

Immunity is a fundamental aspect of animal biology, defined as the host’s ability to detect and defend against harmful pathogens and toxic substances to preserve homeostasis. However, immune defenses are metabolically demanding, requiring the efficient allocation of limited resources to balance immune function with other physiological and developmental needs. To achieve this balance, organisms have evolved sophisticated signaling networks that enable precise, context-specific responses to internal and external cues. These networks are essential for survival and adaptation in multicellular systems. Central to this regulatory architecture is the STAT (signal transducer and activator of Transcription) family, a group of versatile signaling molecules that govern a wide array of biological processes across eukaryotes. STAT signaling demonstrates remarkable plasticity, from orchestrating host defense mechanisms to regulating dietary metabolism. Despite its critical role, the cell-specific and context-dependent nuances of STAT signaling remain incompletely understood, highlighting a significant gap in our understanding. This review delves into emerging perspectives on immunity, presenting dynamic frameworks to explore the complexity and adaptability of STAT signaling and the underlying logic driving cellular decision-making. It emphasizes how STAT pathways integrate diverse physiological processes, from immune responses to dietary regulation, ultimately supporting organismal balance and homeostasis.

## 1. Introduction

Organismic complexity varies widely across the animal kingdom, from simple organisms like *C. elegans*, with thousands of cells, to highly sophisticated mammals like humans, composed of billions [1,2]. In any multicellular organism, immunity is a fundamental feature essential for survival, enabling competition and coexistence within a defined ecosystem [3,4]. Achieving an optimal immune response requires a sophisticated interplay of sensors, processors, and activators, forming highly integrated cellular signaling networks. Beyond immunity, other essential physiological processes—such as metabolism and dietary regulation—depend on similarly intricate and modular signaling circuits that continuously assess internal and external conditions [5,6,7,8]. These networks allow cells to execute precise, context-aware decisions, optimizing the allocation of finite resources within a dynamic environment to maintain adaptation and homeostasis.

Central to our understanding of cellular signaling and cell-to-cell communications is the signal transducer and activator of transcription (STAT) family, a highly conserved and versatile signaling network across eukaryotes [9,10,11,12]. STAT proteins have profoundly shaped our understanding of human physiology and disease, leading to the development of impactful therapies for infectious, inflammatory, and autoimmune disorders, benefiting millions worldwide [13,14,15,16,17]. Yet, a fundamental question remains: How do just a handful of STAT proteins orchestrate such diverse, cell-specific, and context-dependent responses to regulate immunity, metabolism, and homeostasis?

This review explores the evolving concept of immunity and cellular decision-making, focusing on the remarkable complexity and adaptability of STAT signaling in shaping physiological processes that span immune defense and metabolic regulation.

## 2. Overview of Immunity and Immunotolerance

The immune system is fundamental to maintaining the delicate balance between health and disease, enabling organisms to combat infections while preserving homeostasis [3,18]. Immunity and immune tolerance are two sides of the same coin, vital for the survival and well-being of multicellular organisms. Immunity refers to the mechanisms by which the body detects and responds to harmful pathogens, while immune tolerance ensures that the immune system avoids damaging its own tissues and tolerates beneficial foreign agents, such as commensal microbes. These processes have co-evolved to balance defense with self-preservation, avoiding aberrant immune responses that could harm the host [3,18,19].

The origins of the immune system can be traced to ancient life forms. Even single-celled organisms like bacteria exhibit primitive immune-like mechanisms, such as the CRISPR–Cas system, which defends against viral infections [20]. As multicellular life evolved, immunity developed into a sophisticated system, typically categorized into innate and adaptive branches. Innate immunity, evolutionarily conserved across invertebrates and vertebrates, provides a rapid, non-specific response to pathogens using barriers (e.g., skin and mucosal surfaces) and cellular defenses (e.g., phagocytes). It recognizes pathogens through pattern recognition receptors (PRRs) that detect evolutionary-conserved pathogen-associated molecular patterns (PAMPs), representing a mechanism preserved across species [3,21,22,23]. Adaptive immunity, primarily present since the evolution of jawed vertebrates, involves specialized cells like T and B lymphocytes capable of recognizing specific antigens [24]. This branch of immunity is characterized by its memory component, enabling a faster and more robust response upon re-exposure to the same pathogen. Adaptive immunity’s evolution has been pivotal, allowing vertebrates to counter diverse pathogens with high specificity and durability [25,26].

**Figure 1:**
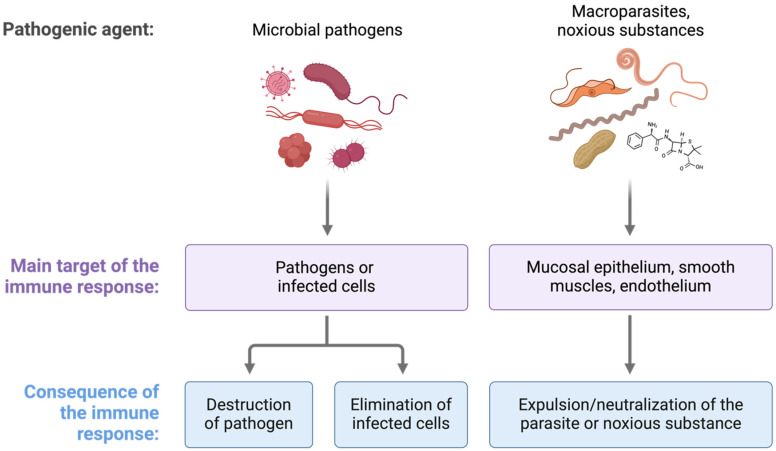
Aim of the immune response. The primary goal of the immune response is to prevent the entry of pathogens and harmful substances while simultaneously detecting, eliminating, and neutralizing noxious substances, pathogens, and infected cells.

The immune system employs a multifaceted response to neutralize and expel noxious substances and parasites, integrating both innate and adaptive mechanisms. Upon the detection of harmful agents, epithelial barriers, mucus secretion, and mechanical actions like coughing aid in their expulsion [27,28]. Innate immune cells, such as macrophages, neutrophils, and eosinophils, recognize PAMPs and release inflammatory mediators, including cytokines and reactive oxygen species, to neutralize threats [29,30]. In parasitic infections, eosinophils and mast cells play a crucial role by degranulating toxic compounds like major basic protein (MBP) and histamine, promoting parasite clearance. Adaptive immunity further refines the response, with IgE antibodies binding to parasites and facilitating their removal via Fc receptor-mediated mechanisms [31,32,33]. Additionally, immune cells like eosinophils can encircle invading parasites, leading to their encapsulation and contributing to chronic infection [34,35]. The immune system neutralizes toxins through antibody-mediated mechanisms, primarily via IgG and IgA, which bind toxins to prevent their interaction with host cells and facilitate their clearance by phagocytes [36,37]. Additionally, immune cells, such as macrophages, degrade toxin-bound immune complexes, reducing their harmful effects [38,39]. To avoid allergens, the immune system relies on mucosal barriers, regulatory T cells, and immune tolerance mechanisms to prevent unnecessary immune activation [40,41,42]. This coordinated defense prevents the entry of pathogens and harmful substances while detecting, eliminating, and neutralizing threats in cases of infection or toxin exposure, thus preventing tissue damage and restoring homeostasis (Figure 1).

A critical feature of immune evolution is the development of tolerance mechanisms, ensuring that the immune system can distinguish between harmful pathogens and self or beneficial agents. For example, regulatory T cells (Tregs) and central tolerance mechanisms, such as T- and B-cell selection, prevent autoimmunity [43,44,45]. Furthermore, immune tolerance extends to commensal microbes that reside on mucosal surfaces and contribute to host functions like digestion and immune regulation. The human gut microbiota, which harbors trillions of bacteria, exemplifies this relationship. Mechanisms like mucus production and antimicrobial peptides maintain microbial balance without triggering harmful inflammation [46,47,48]. The microbiota also plays a crucial role in shaping immune tolerance by promoting immune cell development and tolerance pathways, highlighting a co-evolved interdependence between host and microbes [49].

The interplay between immunity and tolerance is dynamic and essential for maintaining health. Disruptions in this balance can lead to disease. For example, a breakdown in tolerance may result in autoimmune conditions, while excessive tolerance could leave the host vulnerable to infections or cancer. This balance becomes increasingly strained with age, contributing to chronic inflammation and autoimmunity, a phenomenon known as “inflammaging” [4,50]. Immunotherapies, such as checkpoint inhibitors for cancer treatment, illustrate the therapeutic potential of modulating this balance but also underscore the risks, as these treatments can lead to autoimmune side effects by disrupting tolerance [51]. Understanding the intricate relationship between immune activation and tolerance is essential for advancing therapies for autoimmune diseases, infections, and cancer, highlighting the immune system’s dual role in defense and homeostasis.

## 3. The Economics of Immunity

Mounting an immune response is energy-intensive, requiring significant metabolic resources that could otherwise support essential processes such as growth, reproduction, and maintenance. This necessitates a careful balance to ensure that immune responses are effective yet sustainable. Disruptions to this balance can lead to chronic infections, metabolic disorders, autoimmune diseases, cancer, or even death [52,53]. The costs of immunity can be categorized primarily into two types: energy costs and choice costs [54]. Understanding these energy and choice costs provides insights into the sophisticated mechanisms that organisms have evolved to optimize immunity while maintaining health and homeostasis [55].

### 3.1. Energy Costs

These are explicit and reflect the metabolic demands required to fuel immune responses. They can be divided into three main categories:

1. Developmental Costs: The energy invested during hematopoiesis to produce diverse innate and adaptive immune cells. This process ensures the availability of immune cells capable of responding to various pathogens [56,57].
2. Maintenance Costs: Resources needed to sustain immune cells over their lifespan. Even in the absence of infection, immune cells require continuous energy for survival, repair, and readiness [57,58,59].
3. Mounting Costs: The energy expenditure associated with activating immune cells during an infection. This includes pathogen recognition, inflammatory signaling, and the activation and proliferation of immune cells [54,56,57]. Fever, a common immune response, exemplifies the systemic energetic demand, as it raises body temperature to inhibit pathogen replication but significantly increases metabolic rates. If unregulated, these demands can deplete vital resources, threatening survival and emphasizing the importance of precise control mechanisms to balance immune activity with metabolic costs [60,61,62].

### 3.2. Choice Costs

These are implicit trade-offs arising from the body’s allocation of limited resources to prioritize immunity over other physiological functions or prioritizing a specific immune response over another [54,63]. Rooted in evolutionary cost–benefit analysis, these trade-offs require balancing the advantages of robust immune defense against potential downsides, such as metabolic strain or collateral tissue damage [4].

The balancing act is highly context-dependent, varying by pathogen type and tissue. For example, in the gut, where a symbiotic microbiota plays essential roles in digestion and nutrient absorption, an overly aggressive immune response risks disrupting this equilibrium. To maintain homeostasis, the gut immune system often prioritizes tolerance, activating an immune response only when necessary [49]. This selective strategy highlights the complexity of immune regulation, ensuring that responses align with minimizing harm to overall physiological integrity. Balancing these trade-offs is essential for ensuring survival in the face of diverse and dynamic challenges [64].

## 4. The “ART” of Immunity Decision-Making

**Figure 2:**
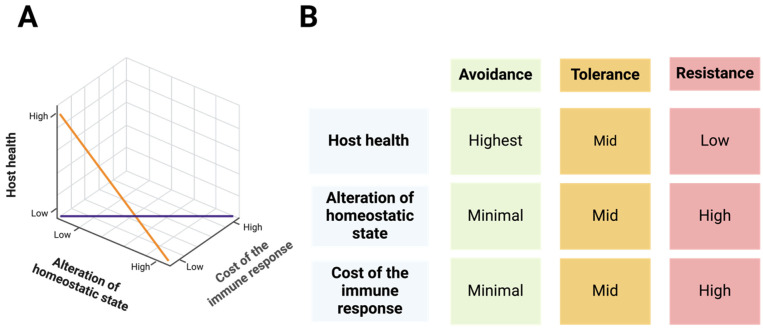
The ART Strategy of Immune Responses. (**A**) An optimal immune response considers three key factors: host health, the cost of the immune response, and alterations to the homeostatic state. (**B**) Based on these factors, immune responses can be classified into three strategies: avoidance, tolerance, and resistance. Resistance, which imposes the highest cost on the host, represents the most resource-intensive strategy. See the text for more details.

To mitigate the high costs of immune activation, organisms have evolved strategies to optimize immune responses, balancing efficiency and resource expenditure [3,19,52,53]. These strategies are encapsulated in the ART framework: Avoidance, Resistance, and Tolerance [65]. Each approach plays a distinct role in immune defense and homeostasis, dynamically adapting to the type and severity of threats as well as the organism’s energy resources (Figure 2).

### 4.1. Avoidance: The First Line of Defense

Avoidance mechanisms prevent pathogens from entering the body and reduce exposure to harmful substances [66,67,68]. This energy-efficient strategy involves the following:

Physical Barriers: Structures such as skin and mucosal membranes serve as shields, while secretions like mucus trap microbes and prevent adherence to epithelial surfaces [69,70,71].

Chemical Defenses: Antimicrobial peptides in the skin and mucosa neutralize pathogens before they can proliferate [72,73,74].

Behavioral Adaptations: Actions such as allergen avoidance further minimize exposure to harmful agents [66,67,75].

By curtailing pathogen entry, avoidance strategies conserve energy that would otherwise fuel inflammation and repair [75]. Additionally, these mechanisms help maintain homeostasis by reducing the risk of tissue damage and systemic inflammation.

### 4.2. Resistance: Active Pathogen Clearance

When pathogens bypass avoidance mechanisms, resistance strategies are activated to detect and eliminate invaders. Innate immunity provides rapid, non-specific responses involving cells like neutrophils, macrophages, and NK cells. Adaptive immunity delivers a slower, antigen-specific response through T and B cells, ensuring pathogen clearance and establishing immunological memory [29,30,76].

While resistance is highly effective, it is resource-intensive, demanding energy for processes like cell proliferation and cytokine production [77]. Moreover, excessive or prolonged resistance responses can lead to collateral tissue damage, as seen in tuberculosis, where chronic inflammation damages lung tissue in attempts to control *Mycobacterium tuberculosis* [78]. Thus, resistance requires precise regulation to avoid unintended harm.

### 4.3. Tolerance: Minimizing Damage

Tolerance mechanisms aim to limit tissue damage and maintain function during infection, even if pathogens are not fully cleared [79,80]. Tolerance prioritizes the integrity of critical processes, such as gas exchange in the lungs or nutrient absorption in the gut, by reducing inflammatory responses [65]. It enables time for tissue repair and the production of anti-inflammatory cytokines (e.g., IL-10) to mitigate the systemic effects of infection [81]. For instance, during malaria, the liver modulates immune responses to minimize systemic damage caused by *Plasmodium* parasites, enabling the host to survive longer under the parasitic burden [79,82,83].

Effective immunity requires a dynamic balance among avoidance, resistance, and tolerance, tailored to the specific context. For example, in acute infections, resistance may take precedence to rapidly neutralize threats. Meanwhile, in chronic infections or sensitive tissues, tolerance often dominates to preserve tissue integrity and physiological functions [84]. This flexibility ensures survival while minimizing energy expenditure and collateral damage. The ART framework highlights the evolutionary ingenuity of immune decision-making, balancing the dual imperatives of defense and homeostasis.

## 5. STAT Signaling: The Mastermind of Immune Cell Decision-Making and Communication

Cytokines serve as the quintessential information transmitters, which regulate communication between immune cells. The balance between pro-inflammatory and anti-inflammatory cytokines is essential for optimal immune responses and preserving homeostasis [85]. The STAT protein family plays a pivotal role in immune regulation, mediating cellular responses to extracellular signals like cytokines and growth factors [86]. The mammalian STAT family consists of seven members, namely, STAT1, 2, 3, 4, 5A, 5B, and 6 [9,10]. STATs orchestrate the integration of innate and adaptive immune functions to combat pathogens, regulate inflammation, and preserve immune tolerance [87,88]. Dysregulated STAT signaling disrupts immune homeostasis, driving conditions such as autoimmunity, chronic inflammation, and cancer, underscoring their significance as therapeutic targets [9,10,14,16].

### 5.1. STAT Signaling in Barrier Defense

In barrier tissues like the skin, lung, and gut, STAT proteins preserve integrity while responding to external challenges.

Skin: STAT3 regulates antimicrobial peptides and wound healing via IL-6/IL-22 signaling, while excessive STAT3 activation contributes to psoriatic inflammation [89]. STAT5 supports Tregs, preventing autoimmunity and inflammation [90,91].

Lung: STAT6 facilitates Th2 responses for parasite defense but drives allergic inflammation in asthma [92,93]. STAT3 aids in pathogen clearance and inflammation resolution but, when dysregulated, contributes to conditions like COPD [94,95,96,97].

Gut: STAT3 and STAT5 maintain mucosal immunity and tolerance. STAT3-driven Th17 cells protect against bacterial infections but are implicated in inflammatory bowel diseases when overactivated. STAT5 supports Tregs, promoting tolerance to dietary antigens and microbiota [98,99,100,101,102,103,104]. STAT6 has been reported to promote gut immune cell function, playing important roles in the clearance of intestinal parasites and allergens [105]. STAT6 plays both protective and detrimental roles in gut epithelial cells by promoting tuft cell development and dysregulating epithelial tight junctions, respectively [106,107,108].

### 5.2. STAT Signaling in Immune Responses and Tolerance

In innate immunity, STAT1 and STAT2 are pivotal in initiating antiviral defenses. Activated by type I interferons (IFN-α/β), they form the ISGF3 complex with IRF9, which induces the transcription of antiviral genes [109,110,111,112]. STAT1 also mediates type II interferon (IFN-γ) signaling, essential for macrophage activation, antigen presentation, and inflammation [113,114,115]. STAT1 is critical for natural killer (NK) cell maturation, cytotoxicity, and response to viral infections. It mediates IFN-γ signaling, enhancing NK cell activation and promoting the transcription of key genes involved in antiviral defense and tumor surveillance [116,117,118]. STAT3 and STAT5 play regulatory roles. STAT3, activated by IL-6 and IL-10, tempers excessive inflammation by suppressing pro-inflammatory cytokine production, thereby preventing tissue damage [119,120,121,122,123,124,125,126]. STAT5, in response to IL-2, supports the proliferation and survival of NK cells, essential for targeting infected or transformed cells [127,128,129].

In adaptive immunity, STAT4 and STAT6 are central to T helper (Th) cell differentiation. STAT4, driven by IL-12, promotes Th1 responses critical for intracellular pathogen clearance via macrophage activation and cytotoxic T cell support [130,131,132]. Conversely, STAT6, activated by IL-4, directs Th2 differentiation, enhancing antibody production and defense against extracellular pathogens like parasites [133,134,135].

STAT3 and STAT5 also influence adaptive immunity. STAT3 supports Th17 cell development, which is crucial for mucosal defense, but is implicated in autoimmunity when overactivated [136,137,138]. STAT5, activated by IL-2, maintains regulatory T cells (Tregs), which suppress excessive immune responses and enforce tolerance to self-antigens, preventing autoimmunity [131,139,140,141,142].

STAT proteins exhibit counter-regulatory roles essential for immune balance. For example, while STAT1 drives pro-inflammatory responses, STAT3 mitigates them to prevent excessive tissue damage. Similarly, STAT5-driven Treg activity counters overactive Th2 responses mediated by STAT6, reducing the risk of allergic diseases while maintaining tolerance. Dysregulation in these pathways can lead to chronic inflammation, autoimmune disorders, or cancer [13].

## 6. The Role of STAT Signaling in Dietary Control and Metabolism

Beyond their classical roles in immunity, the STAT protein family plays a central role in connecting diet, metabolism, and immune function.

### 6.1. STAT Proteins and Immune Cell Metabolism

STAT proteins are critical regulators of immune cell metabolism, supporting differentiation, proliferation, and effector functions. Immune cell metabolism and STAT signaling are tightly interconnected, with specific STAT proteins modulating pathways that influence immune activation, tolerance, and homeostasis. STAT1, activated primarily by interferons (IFNs), orchestrates antiviral and antibacterial responses while promoting inflammatory programs [87,143]. In macrophages, STAT1 drives a metabolic shift to aerobic glycolysis (the “Warburg effect”), ensuring rapid ATP and precursor molecule generation to fuel cytokine production and proliferation [77,144]. This metabolic reprogramming underpins the functions of M1 macrophages, enabling the production of pro-inflammatory mediators like nitric oxide and reactive oxygen species critical for pathogen clearance [145,146]. Concurrently, STAT1 suppresses oxidative phosphorylation (OXPHOS), reinforcing the reliance on glycolysis. This metabolic commitment supports inflammation but must be tightly regulated to prevent chronic tissue damage [147,148].

In contrast to STAT1, STAT3—activated by cytokines such as IL-6 and IL-10—fosters anti-inflammatory and regulatory responses by promoting OXPHOS and lipid metabolism [149]. STAT3-driven metabolic programming supports regulatory T cells (Tregs) and M2 macrophages, key players in immune tolerance and tissue repair [150,151,152]. Tregs rely on fatty acid oxidation (FAO) to sustain mitochondrial function and maintain immune homeostasis [153]. Similarly, STAT3-induced FAO and OXPHOS in M2 macrophages facilitate wound healing and the resolution of inflammation [154,155,156]. The dysregulation of STAT3, however, can impair immune tolerance, leading to chronic inflammatory conditions [157].

STAT5, activated by IL-2 and other growth factors, is essential for T cell proliferation and effector differentiation. It drives glycolysis and OXPHOS to meet the metabolic demands of rapidly dividing effector T cells while ensuring flexibility in nutrient-limited environments. By enhancing glucose uptake and mitochondrial biogenesis, STAT5 supports cytokine production and cytotoxicity, critical for robust immune responses [158,159,160,161].

The interplay between STAT signaling and metabolism ensures that immune cells are equipped to respond dynamically to infections, inflammation, and tolerance needs. The dysregulation of STAT signaling pathways has significant implications in disease [162]. In chronic inflammation, persistent STAT1-driven glycolysis can fuel inflammatory diseases. In immune suppression in cancer, aberrant STAT3 activity promotes tumor immune evasion by reprogramming metabolism in the tumor microenvironment. In autoimmune diseases, shifting STAT signaling from glycolysis to OXPHOS may restore balance in autoimmune disorders [158,160,163,164,165,166]. Targeting STAT-mediated metabolic pathways offers therapeutic potential in cancers, autoimmune conditions, and chronic inflammatory diseases, leveraging the precise regulation of immune metabolism to optimize treatment outcomes [167,168].

### 6.2. The Crosstalk Between STAT Proteins and Dietary Regulation

Nutrients significantly influence STAT signaling, with effects varying based on dietary composition and individual metabolic states. Saturated fats and sugars, prevalent in Western diets, activate inflammatory pathways involving STAT3 and STAT1, contributing to conditions like obesity and insulin resistance [169]. STAT3, responsive to hormones like insulin and leptin, regulates lipid metabolism and inflammation, particularly in response to high-fat diets [169,170]. In contrast, polyunsaturated fatty acids (PUFAs), especially omega-3 fatty acids, inhibit STAT3 phosphorylation, mitigating inflammation and lowering the risk of metabolic diseases [171,172]. These findings highlight how dietary fats exert dualistic effects on STAT signaling, with quality playing a decisive role.

The gut microbiota, heavily influenced by diet, modulates STAT pathways, further linking diet to immune responses [173]. Short-chain fatty acids (SCFAs), products of fiber fermentation, interact with STAT6 to enhance immune tolerance in the gut. High-fiber diets promote SCFA production, reducing inflammation by activating regulatory T cells and supporting immune balance [174]. Polyphenols, found in fruits and vegetables, shape the gut microbiota and inhibit STAT1 and STAT3 activity. These compounds reduce pro-inflammatory cytokines while promoting antioxidant responses, demonstrating their potential to mitigate systemic inflammation [175,176,177].

Caloric restriction (CR) and intermittent fasting (IF) profoundly influence STAT signaling, enhancing metabolic health. CR modulates STAT3 to improve mitochondrial function and stress responses, promoting longevity and metabolic regulation [178]. During fasting, reduced STAT3 activity in adipose tissue and the liver increases fatty acid oxidation and insulin sensitivity, reducing inflammation and enhancing autophagy [179]. STAT5 activity, influenced by growth hormone and IGF-1, decreases during CR and fasting, promoting cellular repair and reducing oxidative stress [180]. This balance underscores STAT proteins’ role in adapting to nutrient availability.

## 7. The “Theory of Relativity” in STAT Signaling

The limited number of STAT proteins necessitates their utilization by multiple signals, with a relative potency in the activation of two or more STATs by a single signal or distinct signals that preferentially activate different STAT proteins. This mechanism enables the fine-tuning of specific and context-aware signaling. A clear example of this is cytokine signaling. Some STATs are activated by relatively few cytokines [181]. For instance, STAT6 is activated exclusively by IL-4 and IL-13, STAT2 primarily by type I interferons, and STAT4 by IL-12, IL-23, and IL-35. In contrast, STAT1, STAT3, and STAT5 (STAT5A and STAT5B) are activated by a broader spectrum of cytokines [182,183,184,185]. Cytokines achieve fine-tuned immune responses through the selective activation of specific STATs. While STAT1, STAT3, and STAT5 are activated by multiple cytokines, each cytokine typically strongly activates only one or two of these STATs. For example, IL-2, IL-7, IL-9, and IL-15 dominantly activate STAT5A and STAT5B, with weaker activation of STAT1 and STAT3 [186]. In contrast, IL-21 predominantly activates STAT3, with more transient activation of STAT1 and STAT5. Moreover, IL-21-mediated STAT3 activation is more sustained, contributing to distinct downstream effects [185,187].

Despite binding similar DNA motifs, STAT proteins elicit distinct gene expression patterns. For instance, IL-21 signaling relies heavily on STAT3 but also critically involves STAT1. IL-21 enhances IFN-γ expression via STAT1, a Th1-dependent effect, whereas STAT3 activation can inhibit these genes. Studies on Stat3-deficient mice and patients with hyper-IgE syndrome further highlight STAT3’s role in modulating gene expression, including its negative regulation of IFN-γ [187]. Cytokines like IL-6 and Fas also fine-tune the balance between STAT1 and STAT3. Fas not only mediates apoptosis but also modulates Th17 and Th1 differentiation. Fas sequesters STAT1, limiting Th1 differentiation while stabilizing Th17 cells in a STAT3-dependent manner. In Fas-deficient cells, IL-6-induced STAT1 activation promotes a Th1 transcriptional program unless STAT1 is also absent. These findings emphasize the importance of regulating Fas levels to balance Th1 and Th17 differentiation [188].

Fine-tuning extends to other cytokine signaling balances, such as IL-2 versus IL-21 or IL-6 versus IL-27. For example, IL-2 promotes Th9 differentiation, while IL-21 inhibits it; the reverse occurs for T follicular helper (Tfh) differentiation. Similarly, Th17 differentiation depends on STAT3 activation by IL-6 and is inhibited by IL-2/STAT5, which interferes with STAT3 binding at the Il17a locus and reduces gp130 expression, thus impairing IL-6 signaling [163,189,190]. IL-27 provides another example of dual STAT activation, using gp130 to activate both STAT1 and STAT3. Unlike IL-6, which promotes Th17 differentiation and suppresses Th1 differentiation, IL-27 promotes Th1 differentiation and inhibits Th17 differentiation [85,181]. Despite these contrasting effects, both cytokines regulate overlapping gene sets via STAT3 [188,191]. The balance between cytokines such as IL-12 and IL-23 also illustrates fine-tuning mechanisms. IL-12 drives Th1 differentiation by activating STAT4, while IL-23 promotes Th17 differentiation via STAT3. These distinct outcomes stem from differences in receptor usage and downstream STAT activation, despite shared components like IL-12Rβ1 [192,193].

Cytokine signals can also cooperate. For example, optimal Th2 differentiation requires IL-2 and IL-4, which activate STAT5 and STAT6, respectively. IL-4-mediated STAT6 drives Th2 differentiation, while IL-2/STAT5 primes cells by promoting chromatin accessibility at the Il4 locus and enhancing IL-4Rα expression [194,195]. Similarly, for Th1 differentiation, IL-2/STAT5 primes cells for IL-12 responsiveness by inducing T-BET and IL-12Rβ2 expression, facilitating STAT4 activation [190]. Overall, cytokine fine-tuning is achieved through the relative potency and interplay of STAT activations, whether through competitive or cooperative mechanisms. This ensures context-specific immune responses, balancing the differentiation pathways of immune cells, and ensuring a tailored and effective immune defense [184].

## 8. The JAK Tip of the STAT Iceberg

### 8.1. Overview of the JAK-STAT Pathway

**Figure 3:**
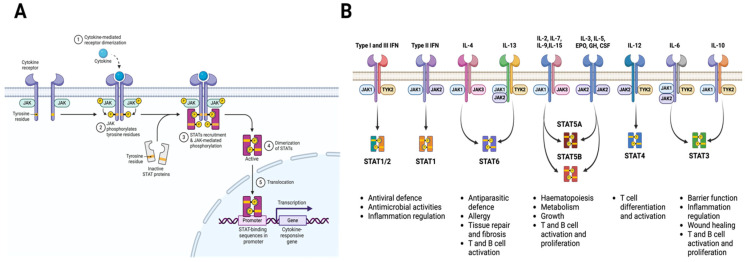
Overview of the JAK-STAT pathway. (**A**) Upon ligand binding—with, for example, cytokines—specific receptors activate one or more JAK kinases, which, in turn, phosphorylate and activate STAT proteins. Once phosphorylated, STAT proteins undergo dimerization and nuclear translocation and drive transcriptional responses. (**B**) Schematic illustration depicting key STAT proteins involved in the signaling pathways of various cytokines and growth factors, along with their major downstream functional roles. See the text for more details. JAK: janus kinase; TYK: tyrosine kinase; STAT: signal transducer and activator of transcription; IFN: interferon; IL: interleukin; EPO: erythropoietin; GH: growth hormone; CSF: colony-stimulating factor.

STAT proteins are highly conserved, versatile signaling molecules found across eukaryotes [196]. They regulate a wide range of cellular processes, including immunity and metabolism, and are notable for their modularity and signaling plasticity [197,198,199]. However, the prevailing paradigm of STAT function remains constrained by the traditional Janus kinase (JAK)-centric signaling model. The JAK family comprises four tyrosine kinases named JAK1, 2, and 3 and tyrosine kinase 2 (Tyk2) [9,10]. In the JAK-STAT framework, STAT proteins are viewed primarily as latent cytoplasmic signaling and transcription factors. Upon ligand binding—with, for example, cytokines, growth factors, or hormones—specific receptors activate one or more JAK kinases, which, in turn, phosphorylate and activate STAT proteins. Once phosphorylated, STAT proteins undergo dimerization and nuclear translocation and drive transcriptional responses (Figure 3A) [87,88]. The JAK-STAT pathway has been central to our understanding of how diverse ligands regulate essential biological processes, including immunity, growth, metabolism, and tissue repair (Figure 3B) [9,10,87]. However, the JAK model falls short of capturing the full spectrum of STAT versatility and context-dependent functionality in immune and metabolic regulation. Emerging evidence has uncovered alternative, non-canonical functions of STAT proteins spanning transcriptional repression, non-nuclear activities, and activities not requiring tyrosine phosphorylation [10,200,201,202].

### 8.2. Tracing Evolutionary Origins

From an evolutionary perspective, STAT proteins predate cytokine receptors and JAKs, with both canonical and non-canonical signaling modes observed in primitive species [203]. Canonical STAT signaling, typically associated with inducible transcription, is found across metazoans. In *Drosophila melanogaster*, the single STAT protein (Stat92E) operates similarly to mammalian STATs, responding to JAK-mediated activation to regulate immunity and development [204,205]. However, earlier-diverging organisms, such as *C. elegans*, possess STAT-like proteins (STA-1 and STA-2) but lack JAKs and cytokine receptors, instead relying on alternative activation mechanisms. For example, STA-2 responds to epidermal injury rather than cytokine signaling [203,206]. Similarly, the slime mold *Dictyostelium discoideum* has multiple STAT proteins that respond to extracellular cyclic AMP (cAMP), rather than cytokines, to regulate developmental and stress-related genes [207,208]. Even plants contain STAT-related GRAS proteins, which contribute to growth and development [209]. Non-canonical STAT signaling, where unphosphorylated STATs function as transcriptional repressors, also appears to be an ancient feature. In *Drosophila*, unphosphorylated Stat92E modulates metabolic and stress-related genes by interacting with heterochromatin protein 1 (HP1), influencing genome stability and tumor suppression [210]. *C. elegans* STA-1 represses antiviral genes in the absence of infection, while *Dictyostelium* STAT proteins regulate gene expression through mechanisms like serine phosphorylation-dependent nuclear export [205,211,212,213,214]. These findings suggest that both canonical and non-canonical STAT signaling evolved early and have been adapted to organismal needs across eukaryotes.

## 9. STAT Signatures: Building a Toolkit for Complex Biological Responses

### 9.1. Key Parameters for STAT-Driven Cellular Functionality

**Figure 4:**
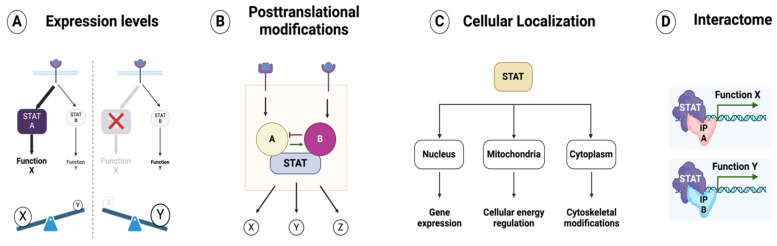
Key parameters for STAT-driven cellular functionality. (**A**) The relative expression of STAT proteins dictates downstream cellular functions downstream of the receptor. (**B**) The interplay between different posttranslational modifications shapes STAT-driven cellular functions. (**C**) STAT proteins exert distinct roles according to their cellular localization. (**D**) Interactions between STAT proteins and other cellular factors regulate downstream cellular responses. See the text for more details. STAT: signal transducer and activator of transcription; IP: interacting protein.

Understanding how vast arrays of signals are interpreted by cells using a limited set of STAT proteins represents both a major challenge and a significant opportunity for developing advanced, more targeted therapeutics that harness the power of STAT signaling [10,13,167,215]. STAT-driven cellular functionality relies on four key parameters: STAT’s expression level, posttranslational modifications (PTMs), cellular localization, and protein interactions (interactome) (Figure 4).

1. Expression levels: The relative expression of STAT proteins and the presence of their isoforms play a crucial role in shaping downstream cellular functions. For instance, IL-21 primarily activates STAT3, which suppresses the expression of the *TBX21* and *IFNG* genes. However, in the absence of STAT3, IL-21 signaling shifts toward STAT1 activation, leading to the upregulation of these genes [187]. This regulatory balance is evident in patients with autosomal dominant hyper-IgE syndrome (AD-HIES, also known as Job syndrome), where STAT3 deficiency due to autosomal dominant mutations results in impaired STAT3 activation, altering cellular responses to IL-21 [216,217]. Beyond overall expression levels, differentially spliced isoforms of STAT proteins further diversify their functions. These isoforms, often characterized by truncations in the C-terminal domain, promote distinct biological outcomes. For example, STAT1α (full-length) and STAT1β (truncated) regulate different subsets of antiviral defense genes, control cell cycle and apoptosis in B cells, and influence NK cell activity and antitumor surveillance [117,218,219,220]. Similarly, STAT3α and STAT3β play distinct roles in cancer progression and inflammation. While STAT3α is widely recognized for its oncogenic properties, STAT3β has emerged as a potential tumor suppressor, underscoring the functional complexity introduced by STAT isoforms [221,222,223].
2. PTMs: PTMs play a crucial role in shaping STAT functionality, with phosphorylation being the most extensively studied, particularly JAK-mediated tyrosine phosphorylation [10,87]. Different phosphorylation events can have synergistic or opposing effects on STAT-driven cellular functions. For instance, STAT1 serine 727 phosphorylation is essential for maximal IFN-mediated STAT1 activity, working in synergy with JAK-mediated tyrosine phosphorylation to enhance antiviral defense [224,225,226,227]. Conversely, STAT1 threonine 748 phosphorylation limits its JAK-mediated tyrosine phosphorylation, thereby promoting inflammatory responses in macrophages [228,229,230]. The interplay between tyrosine and threonine phosphorylation plays a crucial role in the phosphorylation-dependent modulation of STAT-driven immune responses and autoimmunity [229,231]. Additionally, other PTMs—including acetylation, methylation, and ubiquitination—have been shown to fine-tune STAT-mediated cellular functions, further expanding the regulatory landscape of STAT signaling [10,87].
3. Cellular localization: Although STAT proteins primarily function as transcriptional activators in the nucleus, they also exert distinct roles in other cellular compartments, such as the cytoplasm and mitochondria [232]. For instance, STAT3 and STAT5A localize to mitochondria, where they regulate gene expression and remodel cellular metabolism [233,234,235,236]. Additionally, cytoplasmic STAT3 modulates the microtubule network by binding to the C-terminal tubulin-interacting domain of stathmin, thereby counteracting its microtubule-destabilizing activity [237].
4. Interactome: The interactions between STAT proteins and other cellular factors play a crucial role in shaping their functional outcomes. For example, IFN-α and IFN-β promote the formation of the STAT1-STAT2-IRF9 complex, which drives the expression of specific IFN-induced genes like *OAS1*. In contrast, IFN-γ induces STAT1 homodimerization, leading to the activation of distinct IFN-responsive genes such as *IRF1* [238]. Additionally, STAT1’s interaction with NF-κB is critical for cytokine gene expression [201,239,240,241], while the crosstalk between WNT, STAT, and TGF-β pathways (via β-catenin, STAT3, and SMAD3, respectively) shapes context-specific cellular responses [242]. Moreover, STAT proteins interact with the transcriptional coactivators p300/CBP to enhance gene expression by promoting chromatin remodeling and transcriptional activation [243,244,245].

### 9.2. STAT Signature Model

In multicellular organisms, cells serve as the building blocks for complex functions. They can be viewed as microscopic meeting rooms where signaling molecules operate within a hierarchical input–process–output framework [246,247,248,249,250]. Cellular signaling and cell-to-cell communication–despite being inherently complex–consist of a set of common, primitive cell-state changes [251,252]. For instance, while immune cells, epithelial cells, and neurons are distinct cell types, their behaviors are built on similar core cellular responses (e.g., gene expression, secretion, metabolism, cell growth, and cytoskeletal changes) [251,253]. Signaling cassettes act as multimodal percepts, providing individual cells with a high information-processing capacity to interpret both internal and external states. This enables context-specific decision-making within the dynamic environment of a multicellular system [84,254,255].

**Figure 5:**
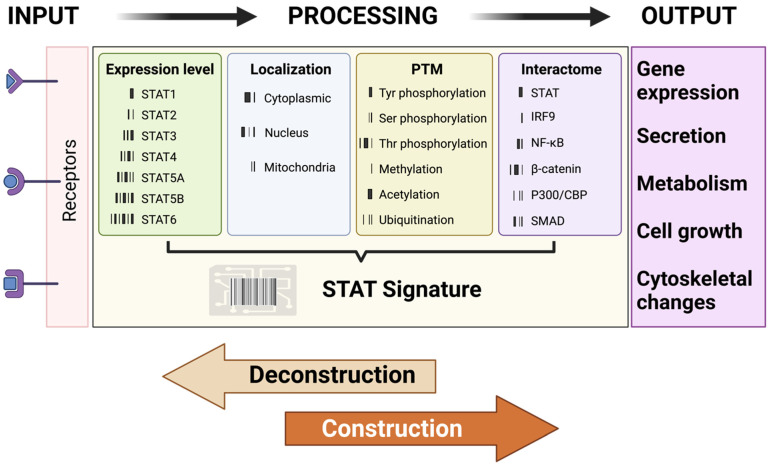
Schematic of the STAT signature working model. Cellular signaling consists of three key components: input (e.g., receptors), information processing (e.g., STAT proteins), and output (e.g., gene expression, secretion, metabolism, cell growth, and cytoskeletal changes). STAT proteins function as multimodal information processors, integrating internal and external signals to drive adaptive and heterogeneous responses critical for multicellular systems. Parameters such as STAT expression, localization, post-translational modifications (PTMs), and protein interactions collectively form “STAT Signatures”, context-dependent barcodes that regulate cell-specific responses based on the cell’s internal and external states. See the text for more details. STAT: signal transducer and activator of transcription; IRF: interferon regulatory factor; NF-κB: nuclear factor-kappa B; CBP: CREB-binding protein; SMAD: suppressor of mothers against decapentaplegic homolog.

Expanding upon these insights, I propose “STAT Signatures” as a dynamic working model of—or, at the very least, a step toward—a more comprehensive framework for understanding STAT signaling, with broader implications for decoding complex cellular signaling networks. This framework positions STAT proteins as multimodal information processors that integrate internal and external signals to enable adaptive and heterogeneous responses essential for multicellular systems. From this perspective, STAT proteins can be viewed as a versatile toolkit. Parameters such as STAT expression, localization, PTMs, and interactome collectively form “STAT Signatures”, context-dependent barcodes that drive composite, cell-specific responses based on a cell’s internal and external states (Figure 5). This provides a dynamic comprehensive model with the potential to provide deeper insights into the complexity of STAT proteins and, more broadly, cellular signaling networks. By deconstructing and constructing the components of STAT signatures, we can gain a better understanding of their functionality and evolution, uncovering the fundamental principles of how cells operate and maintain themselves as self-organizing life forms.

## 10. Conclusions and Future Perspectives

STAT signaling has been instrumental in shaping our understanding of how intracellular pathways enable cells to process information and respond to external cues. STAT-driven cellular responses are highly context-dependent, varying from cell to cell to ensure precise regulation and the efficient use of finite cellular resources.

The proposed “STAT Signature” model represents a shift from the traditional JAK-centric view of STAT proteins toward a more comprehensive framework. This dynamic approach offers new opportunities to unravel the complexities of STAT signaling and decode the molecular logic underlying hierarchical cellular decision-making. By integrating both biological (deconstruction) and synthetic biology (construction) approaches, this framework offers a valuable perspective for exploring the mechanisms governing context-aware cellular responses.

Unraveling how just seven STAT proteins orchestrate diverse cellular responses across various biological contexts will deepen our understanding of the molecular logic driving cellular decision-making and intercellular communication in multicellular systems. Ultimately, these insights could pave the way for groundbreaking therapeutic strategies, leveraging the precision of STAT signaling for targeted interventions in various diseases.

## Data Availability

No new data were created or analyzed in this study.
